# Iron Homeostasis Disorder and Alzheimer’s Disease

**DOI:** 10.3390/ijms222212442

**Published:** 2021-11-18

**Authors:** Yu Peng, Xuejiao Chang, Minglin Lang

**Affiliations:** 1 CAS Center for Excellence in Biotic Interactions, College of Life Science, University of Chinese Academy of Sciences, Beijing 100049, China; pengyu18@mails.ucas.ac.cn (Y.P.); changxuejiao20@mails.ucas.ac.cn (X.C.); 2 College of Life Science, Agricultural University of Hebei, Baoding 071000, China

**Keywords:** Alzheimer’s disease, iron homeostasis disorder, iron homeostasis regulators, β-amyloid, tau, APP, central nervous system, oxidative stress, pathogenesis, genetic intervention

## Abstract

Iron is an essential trace metal for almost all organisms, including human; however, oxidative stress can easily be caused when iron is in excess, producing toxicity to the human body due to its capability to be both an electron donor and an electron acceptor. Although there is a strict regulation mechanism for iron homeostasis in the human body and brain, it is usually inevitably disturbed by genetic and environmental factors, or disordered with aging, which leads to iron metabolism diseases, including many neurodegenerative diseases such as Alzheimer’s disease (AD). AD is one of the most common degenerative diseases of the central nervous system (CNS) threatening human health. However, the precise pathogenesis of AD is still unclear, which seriously restricts the design of interventions and treatment drugs based on the pathogenesis of AD. Many studies have observed abnormal iron accumulation in different regions of the AD brain, resulting in cognitive, memory, motor and other nerve damages. Understanding the metabolic balance mechanism of iron in the brain is crucial for the treatment of AD, which would provide new cures for the disease. This paper reviews the recent progress in the relationship between iron and AD from the aspects of iron absorption in intestinal cells, storage and regulation of iron in cells and organs, especially for the regulation of iron homeostasis in the human brain and prospects the future directions for AD treatments.

## 1. Introduction

The transition metal element iron is the second most abundant metal element in the earth’s crust behind, aluminum. It is also an essential trace element and an important component of metalloprotein for human body [1,2]. Due to its unique chemical reaction characteristics, it plays an important role in maintaining normal physiological function and metabolism, such as oxygen transport, DNA synthesis, iron sulfur cluster synthesis, neurotransmitter synthesis and electron transfer in respiratory chain [3,4,5]. The adult human body contains 3–5 g of iron [2]. In the normal metabolism of the human body, iron ions are absorbed into the blood through the small intestine and transported to the parts of the body requiring iron. Although the body strictly regulates the regulation of iron metabolism, changes with age, genetics and the environment will lead to iron metabolism disorders [6]. The disorder of iron metabolism in the body will catalyze the formation of reactive oxygen species (ROS) through Fenton and other chemical reactions, attack DNA, protein and lipid molecules, and lead to cell damage [7,8]. In recent years, more and more research teams on the pathogenesis of Alzheimer’s disease (AD) have shown that the oxidative stress induced by iron metabolism disorder and the production of ROS are related to the pathological process of AD [7,9]. Alzheimer’s disease is an age-related neurodegenerative disease with clinical symptoms of memory decline, cognitive impairment and learning impairment [10,11,12]. With the increasing human life span, the incidence rate of AD is also increasing, and has become one of the most important fatal diseases [5,13,14]. The pathological features of AD in the brain are the extracellular deposition of Aβ proteins forming insoluble senile plaques and the intracellular accumulation of hyperphosphorylated tau proteins forming neurofibrillary tangles (NFTs), which result in a large degree of neuronal cell death [11,15,16]. Thus far, the main causes and pathogenesis of AD have not been fully clarified. Many research teams have found that there is regional deposition of iron in the brain of AD patients [17,18,19]; treatment with an iron chelator can effectively alleviate the symptoms of AD [9], suggesting that iron metabolism disorder has a close relationship with AD.

This paper reviews the relevant research progress in the field of iron and AD in recent years, focusing on the oxidative stresses induced by normal iron metabolism and its metabolic disorders, especially for abnormal expression of the iron transporters, transferrin receptors, divalent metal transporters, and their relationships with the AD pathological mark proteins, such as Aβ and tau proteins. Relevant contemporary AD treatment measures have also been discussed and prospected. The iron homeostasis on AD provides a theoretical basis for the prevention and treatment of neurodegenerative diseases and an effective drug screening target.

## 2. Physiological Function and Metabolic Process of Systemic Iron

### 2.1. Physiological Function of System Iron

Iron is an essential trace metal element and an important component of metalloprotein [2]. Due to its unique chemical reaction characteristics, iron plays an important role in oxygen transport, DNA synthesis and repair, energy generation and enzyme function, such as the formation of a variety of coordination compounds with organic ligands and redox reactions by the mutual conversion of divalent iron and trivalent iron [3,6,8,20].

### 2.2. Metabolic Process of System Iron

#### 2.2.1. System Iron Absorption

**Figure 1:**
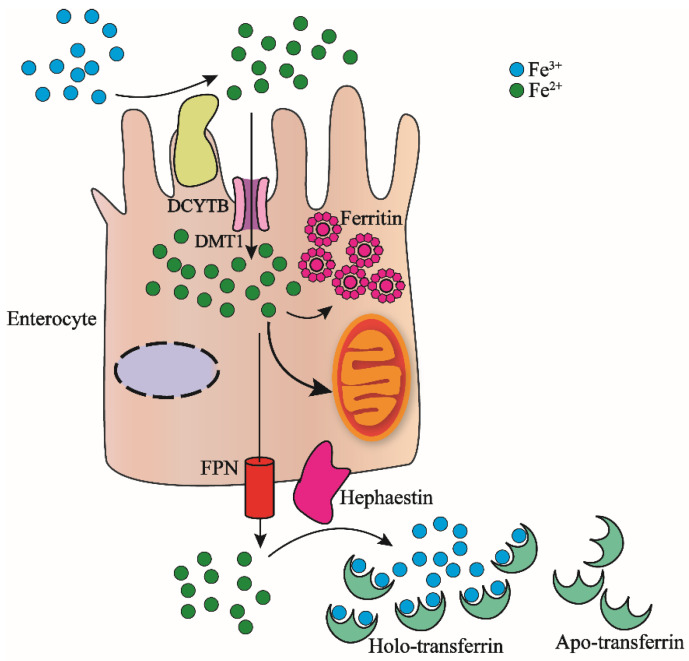
Nonheme iron intestinal absorption and transport by intestinal cells. Food Fe^3+^ is reduced to Fe^2+^ by DCYTB, which binds to the divalent metal transporter DMT1 on the surface of the intestinal cell membrane and transported into the intestinal epithelial cells. The Fe^2+^ that enters the intestinal cells can enter the mitochondria for the synthesis of heme. It can also be oxidized to Fe^3+^ and then stored in ferritin. The excess Fe^2+^ is released from FPN into the plasma and then oxidized to Fe^3+^ by hephaestin. Each molecule of Apo-transferrin in the plasma combines with two Fe^3+^ ions to form Holo-transferrin-Fe. The complex transports iron in the blood to the organs in the body that require iron.

As we know, the adult human body contains about 3–5 g of iron [2], for individuals without blood transfusion, a part of the iron in the system comes from intestinal cells absorbed from food, and the other part comes from macrophages [6,21]. As shown in Figure 1, the absorption of iron from food is Fe^3+^, which is reverted to Fe^2+^ by DCYTB (duodenal cytochrome-b-like protein); then, the divalent metal transporter 1 (DMT1) on the surface of the intestinal cell membrane combines the ferrous iron and transports it into the intestinal epithelial cells [20]. The ferrous iron entering intestinal epithelial cells can be transported to mitochondria for heme molecule synthesis, or oxidized to ferric iron and stored in ferritins [22,23]. Excess Fe^2+^ is released into plasma by FPN (ferroportin) which is located on the basal intestinal cell membrane where it is again oxidated to Fe^3+^ by the same situated hephaestin [24,25,26].

**Figure 2:**
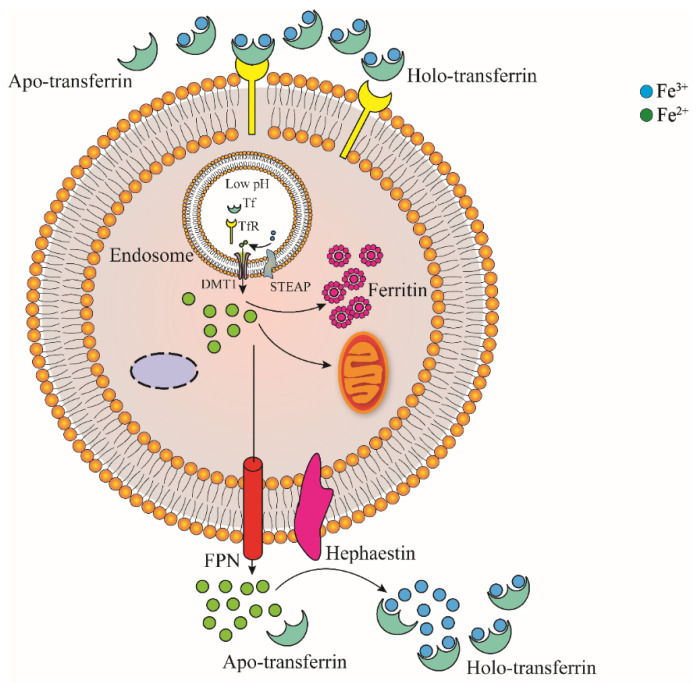
Somatic cell absorption and the transport of iron ions. Fe^3+^ in plasma can bind to Apo-transferrin (Tf), which forms a Tf–Fe complex; then, it is transported through the blood to bind to the transferrin receptor (TfR1) that requires high expression on the surface of iron cell membranes, which enters the iron-requiring cells through endocytosis mediated by clathrin. In the endocytic vesicles, Fe^3+^ is reduced to Fe^2+^ by the six-transmembrane epithelial antigen of prostate (STEAP), After separation in a low-pH environment, Fe^2+^ is released into the cell by the divalent metal ion transporter DMT1. The iron ions in the cell can enter the mitochondria to participate in the redox reaction and can also be stored in the ferritin protein. When the body is in a state of iron limiting, Fe^2+^ can be transported to the outside of the cell through FPN and oxidized by hephaestin to Fe^3+^, and combines with Apo-transferrin to form Holo-transferrin.

As shown in Figure 2, Fe^3+^ in plasma can bind to transferrin (TF), which is transported through blood in the form of TF-Fe complex. The complex then bind to transferrin receptor 1 (TfR1) that highly expressed on the surface of iron demanding cell membrane, and it enters iron demanding cells through clathrin-mediated endocytosis [27,28]. Fe^3+^ in endocytic vesicles is reduced to Fe^2+^ by STEAP (six-transmembrane epithelial antigen of prostate) and released into cells by divalent metal ion transporter DMT1 after separation in low pH environment of endocytic vesicles [29,30,31,32]. In addition, ZIP14 (member of the Zrt/IRT family) was initially identified as a transporter of Zn. In subsequent studies, it was found to be involved in the transport of ferrous iron released from endocytic vesicles into the cytoplasm [1,28,33]. TF and TfR1 separated from Fe^3+^ enter the plasma and are redistributed to the surface of cell membrane to participate in iron transport and the next round of iron absorption, respectively [1]. Fe^3+^ in plasma can also combine with citrate, ATP and ascorbate to form small-molecule complexes [6].

#### 2.2.2. Storage and Loss of System Iron

In the body, iron is mainly stored in liver cells and macrophages. Macrophages phagocytize the aging red blood cells and release the iron ions inside red blood cells; then, the released irons are stored in ferritin proteins in the macrophages [34]. When the body is in a state of iron demand, macrophages secrete ferritin protein into the serum circulatory system; therefore, the concentration of ferritin protein in serum can reflect the state of iron content in the body [35]. Ferritin protein plays an important role in iron storage and antioxidation in cells [36]. Ferritin protein contains two subunits of H-ferritin and L-ferritin, which exhibit ferrous oxidase activity and iron storage function, respectively [37]. Fe^2+^ in cells is oxidized by H-ferritin and stored in L-ferritin. Each ferritin protein can store 4500 iron atoms, which can considerably reduce the cell level of free iron ions and prevent the damage caused by free-iron-induced oxidative stresses; thus, it has antioxidant effects [38]. When the concentration of iron in cells decreases, ferritin protein is decomposed into hemoxanthin by lysosomes. Hemoxanthin and ferritin protein can be detected by Prussian blue staining [39]. In addition to ferritin protein, iron entering the cell can enter mitochondria to synthesize heme, as well as the iron sulfur cluster, and participate in the process of aerobic respiration as a cofactor of mitochondrial respiratory chain protein. It can also combine with some small molecular substances in the cell, such as citric acid, ATP, AMP and pyrophosphate to form an intracellular free iron pool [6,8,40]. The amount of pooled free iron can reflect the change in iron content in cells, which can be detected by some fluorescence techniques [8]. Increasing the pool content will produce harmful substances through redox reactions, causing damage to cells, which could even lead to cell death when it is serious [8,41,42]. Iron entering the blood can also be ingested and utilized by iron cells and iron storage cells. Most of the iron in the blood is used by red blood cells to participate in the transport of oxygen. About 20–30% of the iron is stored in the liver and macrophages, and some iron is involved in the formation of myoglobin, cytochrome and iron-containing enzymes [8].

The normal human body loses about 1–2 mg of iron every day [36,43]. Iron in the body is mainly excreted from intestinal mucosa, skin cells, sweat and urine [4,30,44].

#### 2.2.3. Regulation of Iron in Cells

**Figure 3:**
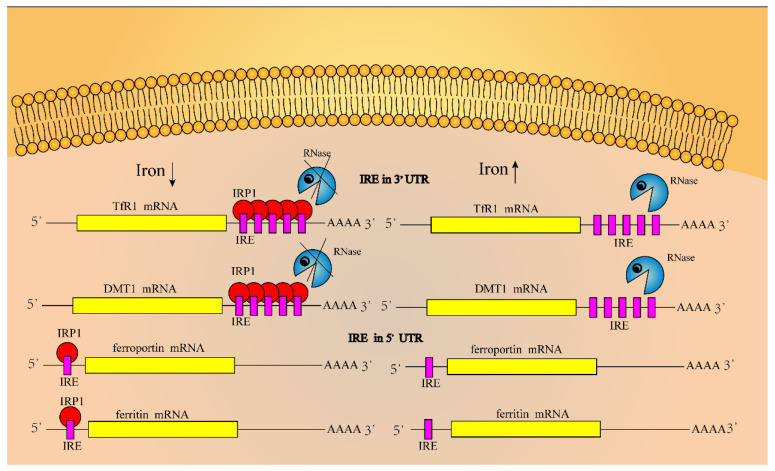
Regulation of iron homeostasis in cells. IREs are located in the 3′-UTR region of TfR1 and DMT1 mRNAs, whereas they are located in the 5′-UTR region of ferroportin and ferritin mRNAs. The combination of IRP and IRE regulates the iron ion concentration in the cell. When the iron ion concentration in the cell is too high, it will induce conformation changes in the untranslated region of mRNAs, making IRPs unable to bind to the IRE region; then, mRNAs of TfR1 and DMT1 are degraded, and the expression level of ferroportin and ferritin increases. On the other hand, iron ions bind to IRP1, and can form iron–sulfur clusters in IRP1 that exhibit cytoplasmic aconitase activity. In contrast, when the iron concentration in the cell decreases, the binding ability of IRP and IRE is enhanced, which leads to a decreased expression of ferroportin and ferritin proteins, and an increased expression of TfR1 and DMT1.

Iron regulatory proteins (IRPs) combine with iron regulatory elements (IREs) in the 3′or 5′ untranslated region of mRNA transcripts of iron-metabolism-related genes to regulate the iron concentration in cells [43,45,46]. The IRE region contains a loop of 5′-cagugn-3′ folded by 30 nucleotides (in which the hydrogen bond formed between G and C stabilizes its structure), without pairing to form hydrogen bonds which will destroy this structure [8,46,47,48]. As shown in Figure 3, IREs are located at the 3′-UTR and 5′-UTR areas of TfR1 and DMT1 mRNAs, and ferroportin and ferritin mRNAs, respectively, although the binding of IRPs to IREs could finely regulate the iron concentration in cells [49]. When the concentration of iron ions in cells is too high, on the one hand, it will induce conformation changes in the untranslated region of TfR1 and DMT1 mRNAs, so that IRPs cannot bind to the IRE region, and those mRNAs are degraded, whereas the expression levels of ferroportin and ferritin proteins are increased; on the other hand, iron ions can bind to IRP1, forming iron sulfur clusters in IRP1 that exhibit cytoplasmic aconitase activity. In contrast, when the concentration of iron ions decreases, the binding ability between IRP and IRE is enhanced, the expression level of ferroportin and ferritin proteins will decrease, and the expression level of TfR1 and DMT1 will increase [48,50,51,52].

Iron regulatory proteins IRP1 and IRP2 are intracellular iron sensors. These are two proteins that are homologous proteins and belong to the iron–sulfur cluster isomerase family [53]. IRP1 can form a cis-aconitase-type iron sulfur cluster (4Fe-4S), which not only determines its functional mode, but also serves as an important regulatory site. IRP1 forms an iron sulfur cluster only when cells are rich in iron, in which IRP1 can display cis-aconitase activity in cytoplasm; however, it reduces the ability of IRP1 to bind to IRE. Low concentrations of iron in cells induce the depolymerization of iron sulfur clusters in IRP1 and enhance the ability of IRP1 to bind to IRE, although the mechanism of iron sulfur cluster depolymerization in IRP1 has not been fully illuminated. In addition, the increase in NO and H_2_O_2_ concentration in cells will activate the activity of IRP1 and promote its binding to IRE [38].

Iron ions and oxygen regulate the synthesis of IRP2 in cells through post-translational mechanisms. IRP2 has lost the activity of aconitase in the process of evolution. The decrease in intracellular iron ions and oxygen concentration promote the synthesis of IRP2 and maintain its stable state. In contrast, the increase in iron ion and oxygen concentration will accelerate the degradation of IRP2. The N-terminal 73 amino acid sequence of IRP2 is characteristic of IRP2. This highly conserved 73 amino acids is encoded by a determined exon and is related to the iron-dependent degradation of IRP2 [8].

#### 2.2.4. Regulation of Iron in the System

Iron ions exported from the intestine are absorbed by the iron-demanding tissues and organs of the body through blood circulation. The liver is the main organ for the regulation of iron balance, which plays an important role in the regulation of whole-body iron balances [34]. The liver produces and secretes the hepcidin hormone [25,54], which is a short polypeptide composed of an 84 amino acid sequence encoded by the HAMP gene and a 25 amino acid sequence hydrolyzed by basic amino acid protein hydrolase [30,34,42,55,56,57,58]. When the iron in the body is in a high-concentration state, hepcidin combines with FPN protein and JAK2 on the intestinal epidermal cell membrane to form a complex, which is phosphorylated before the endocytosis of FPN. FPN is endocytosed into the cell and degraded in the lysosome after ubiquitination to reduce the concentration of iron in the blood [30,46,55,59]. In contrast, when the body is in a state of iron deficiency, hypoxia, inflammation and erythrocyte synthesis, the expression of hepcidin decreases. Some studies have shown that hepcidin can also be produced by other organs and tissue cells, such as the heart, alveolar macrophages and spleen macrophages [42,60,61,62]. In addition to the liver, red blood cells and macrophages participate in the iron metabolism of the body. For example, iron in red blood cells participates in the synthesis of hemoglobin, and macrophages can phagocytize aging red blood cells to release iron; therefore, macrophages could participate in iron circulation when the body is in a state of low iron concentration [42,63].

### 2.3. Roles of Microbiota in Iron Homeostasis and Neurodegenerative Diseases

In mammals, iron ions are absorbed mainly through the duodenum, and there is a strict regulation mechanism for iron ion absorption. Iron ions that are not absorbed into the duodenum end up in the colon cavity, which is home to a host of microbes called the gut microbiome. Iron plays an important role in the growth of intestinal micro-organisms because it plays an important role as a ferritin cofactor in redox reactions, metabolic pathways and electron transport chains of microorganisms. Therefore, the content of iron ions in the colonic lumen will affect the composition, growth and living status of intestinal microbes, and conversely, the changes of intestinal microbes will also affect the health status of the host [64]. A growing number of studies have shown that the gastrointestinal tract and the central nervous system interact through the gut-brain axis, including neuronal, immune and metabolite-mediated pathways. Preclinical and clinical studies have shown that gut microbiome plays a key role in the gut-brain interaction, and that disturbances in the composition of gut microbiota are associated with the pathogenesis of neurological diseases, especially the neurodegenerative diseases [65]. Maternal immune activation (MIA) increases the risk of autism spectrum disorder (ASD) in offspring. Dysregulation of microorganisms is associated with ASD symptoms. In lipopolysaccharide (LIP) -induced MIA progenies, MIA progenies exhibited an abnormal brain-gut-microbiome axis compared with that of the control progenies, which were characterized by social behavioral deficits, anxiety-like and repetitive behaviors, low myelination, and ASD-like microbiome [66]. Studies have shown a potential link between host microbiome (such as gut and oral bacteria), neuroinflammation, and dementia, which may be caused by bacterial invasion of the brain due to barrier leakage, toxin and inflammation factor production, or indirectly by modulating immune responses, and moreover, the composition of microbiota affected the deposition level of Aβ in the cerebral cortex of APP/PS1 mice [67], suggesting a critical role of iron in these processes.

## 3. Brain Iron Metabolism

### 3.1. Brain Iron Absorption

The brain is composed of neurons and glia. Ferritin is also the main iron storage protein in neurons, and neuromelanin has been found to storeiron ions for a long time. In glial cells, astrocytes and microglia synthesize L-ferritin to store iron ions, and L- and H-ferritin are expressed in oligodendrocytes [68]. Cells in the CNS are not in direct contact with nutrients, including iron ions. The blood–brain barrier (BBB) and blood–brain spinal cord barrier (BBSCB) separate the CNS from the system circulation. BBB is a special structure, which is composed of auxiliary feet of capillary endothelial cells, peripheral skin cells and astrocytes, and it strictly regulates the substances entering the CNS [69,70]. The hydrophobic BBB prevents the hydrophilic holo-TF from entering the nervous system. Holo-TF must pass across the BBB through the brain capillary endothelial cells. Holo-TF in the blood circulation binds to the TF receptor TfR1 on the luminal surface of the brain capillary endothelial cells and enters the cells. The FPN on the abluminal surface transports ferrous iron out of the capillary endothelial cells, where Fe^2+^ are oxidized to Fe^3+^ by ceruloplasmin (CP) [71,72]. CP is expressed in astrocytes and promotes the transport of FPN-exported ferrous iron [24,73]. The binding of iron ions into intercellular fluid and cerebrospinal fluid is secreted by nerve cells, especially TF, synthesized and secreted by oligodendrocytes, and choroid plexus cells, which diffuse through brain parenchymal tissue and bind to the TfR1 receptor on the surface of nerve cell membranes. After releasing iron ions, apo-TF enters the blood circulation through arachnoid villi [71,74]. FPN is regulated by hepcidin in the system, although the source of hepcidin in the brain is unknown. It may enter the brain across the BBB for iron metabolism regulation [68].

### 3.2. Brain Iron Regulation

The regulation of brain iron homeostasis at the cellular level involves IRPs regulating the expression of related proteins [9,75,76]. The decrease in the IRP2 expression level will lead to the imbalance of brain iron, but it has little effect on myelin iron. Mutations in genes controlling brain iron homeostasis will lead to the disorder of brain iron metabolism and affect the synthesis of myelin. It is unclear whether hepcidin plays a key role in the mediation of brain iron metabolism; whether hepcidin is synthesized in the brain or passes through the BBB after its synthesis in the liver has not been revealed. Recent results show that inflammation activates microglia and promotes the release of hepcidin by astrocytes in the model of signal cascade between inflammatory cells; this signal prevents the release of iron ions in neurons and eventually leads to neuronal death. At the same time, it will also lead to the release of anti-inflammatory and pro-inflammatory factors. Normal human microglia are not activated, and there is no intercellular signal cascade [36,72,77].

### 3.3. Brain Iron Accumulation and Toxicity

Iron ions accumulate in the brain with age [9,78,79]. Iron ions mainly bind to ferritin protein and substantia nigra [80,81,82]. The accumulation of iron ions can induce neurotoxicity through different mechanisms. The excessive accumulation of iron ions will increase the permeability of the BBB, induce inflammation, affect the redistribution of iron ions in the brain, and then change brain iron metabolism [47]. Iron ions can act as both electron acceptors and electron donors; therefore, when iron ions accumulate in the brain, they will produce reactive oxygen free radicals through Fenton and Haber–Weiss chemical reactions [41,83,84]. Free radicals are highly active substances, which may promote protein oxidation, membrane lipid peroxidation and nucleic acid modification. When the levels of ROS exceed the antioxidant capacity of organelles, this will induce oxidative stress and damage neurons [38,85,86], leading to tissue degradation in severe cases.

Fe^2+^ + H_2_O_2_ → Fe^3+^ + OH^•^ + OH^−^ (Fenton)

Fe^3+^ + O_2_^•^^−^ → Fe^2+^ + O_2_

O_2_^•−^ + H_2_O_2_ → O_2_ + OH^•^ + OH^−^ (Haber–Weiss)

## 4. Iron Metabolism and AD

### 4.1. Effect of Iron Metabolism Disorder on AD

AD is the most common cause of dementia, which is characterized by impaired cognitive function and decreased ability of learning, memory and reasoning [24,87]. It was originally described by Dr. Alois Alzheimer, a German doctor. Patients with this kind of disease exhibit strange behavioral symptoms, memory loss and motor loss. Its histopathological characteristics are amyloid plaques deposited outside the cells, and the excessive phosphorylation of tau protein related to the cytoskeleton which forms neurofibrillary tangles in the cells [88,89,90]. With the increase in age, iron ions in the brain tend to accumulate, especially in the cortex, globus pallidus, red nucleus, dentate nucleus and substantia nigra; however, the related molecular mechanisms are not clear at present [9,74,79]. The emerging evidence shows that iron with high redox activity is related to the deposition of amyloid plaques and the formation of nerve fiber tangles, suggesting it may be one of the main causes of AD [91,92,93,94].

The postmortem brain anatomy of AD patients showed that there was more Aβ deposition and neurofibrillary tangles in the hippocampal region of the patients [95,96,97]. Moreover, by detecting the level of antioxidant protein in the hippocampus and amygdala, the level of oxidative stress in these two regions was found to be much higher than other regions. Moreover, the oxidative stress caused by iron accumulation will enhance the activity of IRP1, resulting in the enhancement of iron absorption through TfR1 and the increase in intracellular free iron level by reducing the concentration of ferritin-H and ferritin-L, which further enhances intracellular oxidative stress [93,98]. Based on magnetic resonance imaging (MRI) technology [99], it was found that iron accumulation may further lead to the deposition of Aβ amyloid and the formation of neurofibrillary tangles in the brain of AD patients. Considerable studies have shown that iron metabolism disorder can affect Aβ misfolding and tau hyperphosphorylation, and the resultant oxidative stress and metal toxicity of iron ions may lead to AD [100,101,102,103].

**Figure 4:**
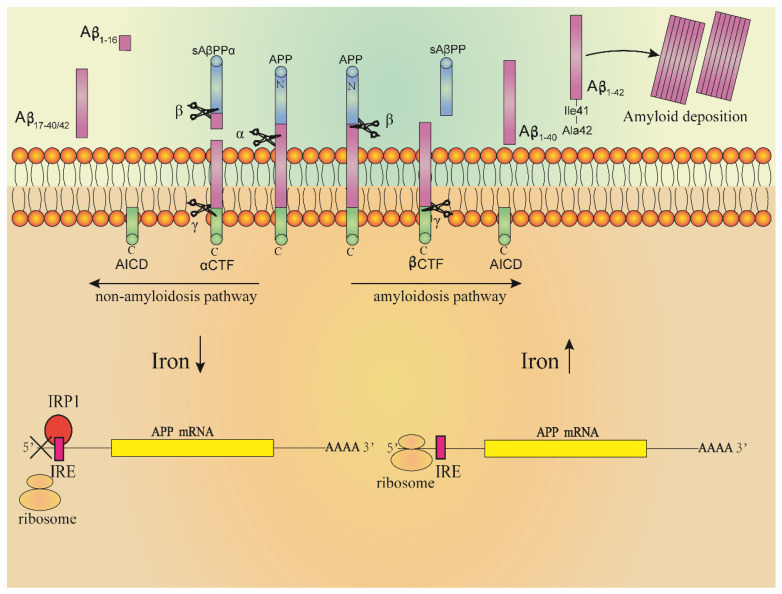
High concentrations of iron in neurons induce Aβ formation. The 5′-UTR region of APP mRNA has an 11-base loop called IRE. The combination of IRPs and IRE regulates the synthesis of APP. The high concentration of iron in the cell will combine with IRP1 to form iron–sulfur clusters, and make IRP1 lose the ability to bind to IRE. At the same time, high concentrations of iron will also induce conformational changes in the IRE region of APP mRNA, which increases the expression of APP; in contrast, when the iron concentration in the cell is at a low level, IRP1 will bind to the IRE of APP mRNA, resulting in the decreased production of Aβ_42_. Aβ_1–42_ aggregates to form amyloid plaques.

Even more evidence supports a key role of ROS and RNS (reactive nitrogen species) in leading to AD, which are toxic and related to the formation of oxidative stress in the brain of AD patients [104]. The oxidative stress was more obvious with the increase in iron concentration, and the oxidation of protein, lipid and DNA in Aβ aggregation area was more significant [105,106]. The free radicals produced at regions of Aβ aggregation will destroy the adjacent neurons, resulting in a decline in cognitive and memory functions. The accumulation of tau protein in neurofibrillary tangles is also related to the induction of heme oxygenase-1 (HO-1). Overexpression of HO-1 can lead to the increase in iron content and accumulation of tau proteins in the mouse brain. In AD patients or patients with slight cognitive impairment, the concentration of HO-1 in the hippocampus and frontal cortex increased [86,107,108]. Increased levels of iron-bound melanin transfer protein were detected in the serum of AD patients, indicating that there may be abnormal binding of iron in the brain of AD patients. It was also found that iron ions accumulated in regions of Aβ deposition and neurofibrillary tangles formed by hyperphosphorylation of tau protein, and which were distributed in hippocampus, parietal cortex and motor cortex [93,106,109,110,111,112]. The Aβ amyloid is a segment of amyloid precursor protein (APP) cleaved by secretory enzymes [113]. APP is a transmembrane protein mainly expressed in the nervous system. At present, the physiological function of APP is not fully understood, and it may play a role in brain development, memory and synaptic plasticity [114]. In nerve cells, the concentration of iron ions regulates expression of the APP gene. The mechanism is shown in Figure 4. There is a loop ring formed by 11 bases in the 5′-UTR region of APP mRNA, which is called IRE. IRPs combine with IRE to regulate the synthesis of APP. High concentrations of iron in cells will combine with IRP1 to form iron sulfur clusters; at the same time, high concentrations of iron will also induce conformational changes in the IRE region of APP mRNA, increasing the expression of APP. In contrast, when the cell iron concentration is at a low level, IRP1 will bind to IRE and the expression of APP will decrease [97,106,115]. Under the action of different secretory enzymes in nerve cells, APP can undergo two different cleaving pathways, including the amyloidosis pathway and non-amyloidosis pathway. In the normal physiological state, APP is cleaved through the non-amyloidosis pathway, in which APP is firstly cleaved by α secretory enzyme, producing a segment called sAβPPα; then, the fragments undergo β and γ secretase cleavage to form non-toxic fragments of P3, Aβ_16_ and Aβ_17–40/42_, respectively. The high concentrations of iron in cells promote the cleaving of APP through amyloidosis pathway, in which APP undergoes β and γ secretases cleavage to form Aβ_1–40_ and Aβ_1–42_ fragments. The Aβ_1–42_ fragment is precipitated by Ile41, and the three histidines at its N-terminal can combine with Fe^2+^ to induce oxidative stress, resulting in Aβ_1–42_ damage to cells at deposition [93,106,109,110,111,115].

In addition, the deposition of Aβ_1–42_ can induce the hyperphosphorylation of tau protein, although the specific mechanism is not clear. At the same time, it can also lead to the disorder of energy metabolism, the activation of immune cells and the disorder of normal function of nerve cells, resulting in cell damage and death [50]. NFTs formed by the hyperphosphorylation of tau proteins and the combination of cytoskeleton mean that the cells are unable to maintain their normal structure. Many neurons in AD patients are affected by NFTs. A large number of NFTs were found in the hippocampus of patients with AD, and the hippocampus participates in the processing of experience and precedes the storage of permanent memory. In the early stages of AD, the clinical manifestations are the decline of learning ability, the ability to form new memory and the memory storage ability. At the same time, the basal forebrain, which provides the innervation activity of cholinergic neurons for the cortex, will also be affected, resulting in the reduction in cholinergic neurotransmitters. Generally, cholinergic enzyme inhibitors can be used to treat the reduction in cholinergic neurotransmitters. A Canadian butylcholinesterase inhibitor exhibited good performance for the treatment of AD symptoms. In clinical treatment, it has been shown that this drug is suitable for the improvement of mild and moderate AD symptoms [116].

### 4.2. Relationship between Iron-Homeostasis-Related Proteins and AD

Oxidative stress can lead to neuronal damage; it has been observed that the disorder of iron metabolism and the expressional change in iron regulatory proteins in the iron metabolism pathway could lead to the accumulation of iron ions in the brain and induce oxidative stress, resulting in the damage of neurons [107]. Many experimental results have showed that iron accumulation in the brain of AD patients is one of the sources of brain oxidative stress, and this has a close relationship with the disorder of brain iron metabolism and some key iron homeostasis regulators, such as ferritin protein, transferrin protein, FPN, etc. [100].

#### 4.2.1. Apolipoprotein E and AD

Apolipoprotein E (ApoE) is involved in the transport of cholesterol and other substances from the brain to the blood, including the discharge of Aβ protein from the brain to the blood. ApoE has three different conformations, which are encoded by *ApoE2*, *ApoE3* and *ApoE4* genes [117]. These three conformations are due to the differences in amino acid composition, resulting in differences in the structure, binding properties and multiple functions of lipoproteins. Among the three conformations, ApoE4 can lead to AD [18,118,119,120,121,122]. It can be seen from the extant literature that high concentrations of iron in cells will induce oxidative stress and cause damage to lipids, proteins and nucleic acids. Among them, lipid peroxidation will induce the production of 4-hydroxynonenal (4-HNE) molecules with high activity and neurotoxicity. It can combine with cysteine residues, lysine residues and histidine residues to reduce its damage to other molecules. Compared with ApoE2 and ApoE3, ApoE4 lacks cysteine amino acid and cannot clear HNE, resulting in the oxidative modification of proteins in neurons and neuronal death, increasing the risk of AD [123,124].

#### 4.2.2. Ferroptosis and AD

Ferroptosis is an iron-dependent programmed cell death, which can lead to many diseases [125]. Ferroptosis was first described by Dixon in 2012, and is characterized by the accumulation of lipid reactive oxygen species. The experimental results show that GPX4 knockout mice exhibit neuronal necrosis, which will become more serious due to the lack of vitamin E (iron death inhibitor) in food. In contrast, inhibiting iron death can effectively improve the symptoms of AD. GPX4 is an anti-peroxidase that inhibits lipid peroxidation [101,126]. Moreover, lipid peroxidation products and 4-HNE in the AD brain have been significantly increased, indicating that ferroptosis will increase the risk of AD [127,128,129]. Iron induces oxidative stress, directly affecting lipids, DNA and proteins. Lipid peroxidation and iron metabolism disorder and accumulation in AD brain are also necessary conditions for ferroptosis [130]. In addition, iron ions interact with Aβ and Tau to induce ROS, which also leads to ferroptosis [101,129].

#### 4.2.3. Iron Homeostasis Key Regulators and AD

Through the utilization of Western blot technology, researchers have found that in comparison with ferritin protein in the normal brain, the expression levels of ferritin protein in the brains of AD patients were increased significantly, including L-ferritin and H-ferritin proteins [36]. The ELISA results showed that the concentrations of H-ferritin and L-ferritin in the hippocampus of AD patients were three times higher than those in normal human brains. Moreover, the increases in H-ferritin and L-ferritin protein concentrations were not consistent with the increase in iron concentration, which was about 50% of the increase in iron concentration. Compared with the normal brain, the expression levels of ferrous oxidase CP increased significantly [131]. Results obtained from immunohistochemical experiments showed that the expression levels of transferrin proteins in the AD brains were also found to be increased compared with those in the normal brain [100].

However, by using Weston blot technology, it was found that the expression levels of DMT1 and FPN decreased in the AD brains compared with those of normal human brains. Due to the abnormal expression of genes related to iron metabolism, iron accumulates in AD brain and induces oxidative stress, which may damage brain neurons [36,131].

#### 4.2.4. Furin and AD

Furin is associated with iron and Aβ metabolism [132]. Low concentrations of iron enhance furin enzyme activity, whereas high concentrations of iron reduce furin enzyme activity. Furin can enhance the activity of α secretory enzymes, and high concentrations of iron in cells reduce furin enzyme activity, leading to the amyloidosis pathway of APP cleaving. Recent experimental results also showed that the expression levels of furin mRNA in the brain of AD patients are lower than those of normal human brains [9,110,133].

## 5. Strategies for Treating AD

### 5.1. Iron Chelation in the Treatment of AD

Iron chelation strategy is the most direct method for limiting and redistributing iron in the system. At present, the most commonly used chelating agents are deferoxamine, deferrone and ferrite [2,134,135]. Deferoxamine is a chelating agent, recently found to exhibit good clinical manifestations. Although these chelating agents can improve the symptoms of AD caused by iron excess to a certain extent, they can also have toxic effects on the human body, such as allergic reactions, liver and kidney failure, etc. [1,136].

### 5.2. Regulating Iron Metabolism Pathway Proteins to Improve AD Symptoms

Fursultiamine is a small molecular substance called thiamine tetrahydrofuran disulfide, which can bind to cys326 amino acid residues of brain FPN and protect hepcidin from the endocytosis of FPN, thus improving the efflux of brain iron through this ferrous transporter. However, fursultiamine has limited functions in the body, because it can be quickly converted to ammonium sulfate, resulting in reduced iron contents in the body [137].

The anti-ferroportin antibody ly2928057 was successfully tested *in vitro*, and it has also been tested for its potential to effectively reduce iron concentrations in vivo by interfering with the potential regulatory mechanism of hepcidin. The specific mechanism is to regulate the BMP6 (bone morphogenetic protein 6)–SMAD signal pathway and prevent the binding of BMP6 to its receptor BMP6R [1,138]. Another way is to block the phosphorylation of SAMD with doxomorphine, so as to reduce the production of hepcidin induced by BMP6R [139]. The body has its own regulatory mechanism; therefore, the treatment of FPN or hepcidin interference is a great challenge, which is not conducive to long-term treatment.

Similar to glutathione peroxidase, ebselene, a drug containing selenium, also exhibits antioxidant effects. This drug can inhibit the absorption of iron ions through DMT1; however, it can cause cardiomyopathy [140]. Recent studies have shown that pyrazole derivatives and benzyl isothiourea have inhibitory effects on DMT1 both in vitro and in vivo [141].

### 5.3. Antioxidant Therapy Improves AD Symptoms

Brain iron excess induces oxidative stress through Fenton chemical reactions, which cause damage to protein, lipid and DNA [7], and lead to ryanodine-receptor-mediated calcium release under the stress, resulting in neurotoxicity [105]. Small molecular substances have been designed for ROS scavenging at fixed sites. These kinds of antioxidants enter the mitochondrial matrix driven by the mitochondrial intimal potential to scavenge active free radicals in the matrix [142]. In addition, antioxidants in food, such as tea polyphenols, can effectively improve AD symptoms through scavenging oxygen free radicals, chelating iron ions and their anti-inflammatory effects [143,144,145]. Other native neuroprotective compounds or species include resveratrol, curcumin, pinocembrin, caffeine, the combination of Panax ginseng, ginkgo biloba, crocus sativus [146,147,148,149,150]. The anti-inflammatory and antioxidant properties of catechins in tea have been reported in vivo and in vitro, with potential for the prevention of AD symptoms [151].

Acetylcholinesterase inhibitors (AChEIs) have also been found to exhibit antioxidant effects. In 2010, Sinem et al. showed that ACHEIs can reduce the levels of lipid oxidation, blood markers and nitric oxide in AD patients [152]. ACHEI is the main drug for the treatment of AD, but it also has certain limitations [153].

## 6. Conclusions and Prospect

Iron is a rich metal element in the earth’s crust. The unique redox properties of iron allow for efficient electron transfer, which is beneficial to many diverse biological reactions [154]. However, when iron metabolism in the body is unbalanced, such reactive properties of iron may also promote the generation of ROS, which will lead to the excessive accumulation of iron ions in the body [155,156]. As a result, there are fine regulatory mechanisms for iron absorption, storage and distribution in organisms. The excessive accumulation of iron induces oxidative stress reactions, which, in large doses, can be damaging to intracellular systems, including the tissues and organs of the body. Moreover, iron plays an important role in the formation of a myelin sheath in the brain and aerobic respiration in mitochondria. When brain iron metabolism is disordered, iron will be enriched in different regions of the brain, and the enriched iron will cause oxidative stress, mediate APP undergoing the amyloidosis pathway, and finally lead to the development of AD. In AD, oxidative stress caused by brain iron accumulation promotes the deposition of amyloid protein and the hyperphosphorylation of tau, which causes damage to neurons, resulting in declines in motor, cognitive and memory functions, etc. [133]. Although using iron-chelating strategies has achieved some positive results for improving the symptoms of AD, there is still much research needed in order to translate the research into practice for the clinical treatment of AD.

Nevertheless, there have been few studies on iron-reducing strategies in AD patients through genetic methods, and excessive emphasis has been put on the amyloid-reducing strategies, which have been disappointing thus far. Given that more and more iron-chelating compounds have potential disease-improving effects, as well as the availability of biomarkers of iron load in MRI and cerebrospinal fluid, there is considerable room for exploring this type of treatment to avoid its side effects as far as possible. In addition, genetic studies on the regulation of some key genes in iron homeostasis in model animals have shown potential for more effective and precise treatment [14,157].

Furthermore, AD is characterized by the progressive dysfunction and death of thecortical and hippocampal neurons; the main hypothetical mechanisms are the hyperphos-phorylation of tau protein to form NFTs and the deposition of Aβ protein to form SPs [158,159]. However, a large number of clinical trials of drugs based on these two hypotheses all over the world have ended in failure; there is currently no effective treatment method. In addition, these two assumptions are facing increasing challenges [160,161]. In fact, the involvement of iron in the pathogenesis of AD has been widely accepted. Iron not only aggravates the accumulation of toxic Aβ and hyperphosphorylated tau, but also directly induces neuronal oxidative damage [162]. Considering the particularity and importance of iron role in the process of ferroptosis, it is essential to uncover how does ferroptosis play in the molecular pathophysiology of AD in the future research, which may provide new insights into the disease [163,164] and new ideas for the treatment [101]. Combing with the recent finding of a potential link among iron, host microbiome and AD, therefore, by deeply studying the mechanism of iron metabolism in the body and brain, it is expected to find new effective targets and therapeutic measures to improve or cure the disease.

## Data Availability

Not applicable.

## Abbreviations

AD	Alzheimer’s disease
CNS	central nervous system
ROS	reactive oxygen species
NFTs	neurofibrillary tangles
DCYTB	duodenal cytochrome-b-like protein
DMT1	divalent metal transporter1
FPN	ferroportin
TF	transferrin
TfR1	transferrin receptor 1
STEAP	six-transmembrane epithelial antigen of prostate
IRPs	iron regulatory proteins
IRE	iron regulatory element
BBB	blood–brain barrier
BBSCB	blood–brain spinal cord barrier
CP	ceruloplasmin
MRI	magnetic resonance imaging
RNS	reactive nitrogen species
HO-1	heme oxygenase-1
APP	amyloid precursor protein
ApoE	apolipoprotein E
4-HNE	4-hydroxynonenal
BMP6	bone morphogenetic protein 6
AChEI	acetylcholinesterase inhibitor
